# 

**Published:** 1893-06-10

**Authors:** 


**OUR NEW OFFICES**
428, Strand, W.C., will henceforth be the Office of this Journal.

**Notices of Births, Deaths, and Marriages,** are inserted in this column at a charge of 2s. 6d. for an announcement not exceeding four lines.

### NOTICE TO CORRESPONDENTS.

All MS., Letters, Books for Review, and other matters intended for the Editor should be addressed The Editor, The Lodge, Porchester Square, London, W.

The Editor cannot undertake to return rejected M.S., even when accompanied by stamped directed envelope.

**Notice to Advertisers and Subscribers.**

All Advertisements, Orders for Copies of The Hospital, and Business Communications should be addressed to The Publisher, *not to the Editor*, The Hospital, 428, Strand, W.C.

The Cost of Subscription, post free, is as follows:—Per week, 2½d.; per quarter, 2s. 6d.; per half-year, 5s.; per annum, 8s. 6d. Foreign:—Per annum, 10s. 8d.; payable, in all cases, in advance.

**"Burdett's Hospital Annual," 1893.**

Fourth year of publication. 700 pp. Boards, gilt lettering. A most complete guide to British, Colonial, Indian, and American Hospitals, Dispensaries, Nursing and Convalescent Institutions, and Asylums. Price 5s. Published by The Scientific Press (Limited), 428, Strand, W.C.

**NOTICE.—The Index for Vol. XIII. (ending March 1893) is on sale at the Office of this Journal, price 2½d., post free.**

### BOOKS RECEIVED.

HENRY KEMPTON.

"The Health Resorts of Europe." By Thomas Linn.

H. K. LEWIS.

"Treatment of Constitutional Syphilis." By Dr. Ziemssen.

"The Sanitary Inspector's Handbook." By Albert Taylor.

LONGMANS AND CO.

"Rural Hygiene." By George V. Poore, M.D.

CHARLES GRIFFIN AND CO.

"Year-Book of the Scientific and Learned Societies of Great Britain and Ireland. 1893."

"Outlines of the Diseases of Women." By John Phillips, M.D.

"A Medical Handbook for the Use of Practitioners and Students." By R. S. Aitchison, M.B., C.M., F.R.C.P.E. Price 8s. 6d.

J. PCRTER, BEDFORD.

"John Howard, the Prison Philanthropist."

CLAUDIUS ASH AND SONS.

"Notes on Anæsthetics in Dental Surgery." By Arthur S. Underwood, M.R.C.S., and C. Carter Braine, F.R.C.S.

SWAN SONNENSCHEIN.
"The Epileptic and Crippled Child and Adult."
"The Maybrick Case." By Dr. Helen Densmore. 1s.

P. BLAKISTON AND CO., PHILADELPHIA, U.S.A.
"Lectures on Mental Diseases, Designed Especially for Medical Students and General Practitioners." By Henry Putman Stearns. $3.

JOHN MURRAY.
"The Physiology of the Senses." By John Gray M'Kendrick, M.D., and William Snodgrass, M.A., M.B.

MACMILLAN AND CO.

"The Soil in Relation to Health." By H. A. Miers, M.A., F.G.S., F.C.S., and R. Crossley, M.A., D.P.H.

HENRY RENSHAW.

"Popular Information Concerning Infectious Diseases." By Horace Sworder, L.R.C.P.Lond.

J. AND A. CHURCHILL.

"Obstetric Aphorisms." By Joseph Griffiths Swayne, M.D.

"The English Baby in India." By Mrs. Howard Kingscote.

CURTIS PUBLISHING COMPANY, PHILADELPHIA.

"A Baby's Requirements." By Elizabeth Robinson Scovil.

BLACKIE AND SON.
"Chemistry for All." By W. Jerome Harrison, F.G.S., and Robert J. Bailey.

BURROUGHS, WELCOME, AND CO.

"The Travellers' A B C Medical and Surgical Guide."

HORNE AND SON, WHITBY.

"Horne's Guide to Whitby."

E. W. ALLEN.

"The New Priesthood." By Ouida.

**Periodicals and Pamphlets.**—The Belfast Health Journal; The Verulam Review; The Engineering Magazine; The Medical Chronicle; The Dublin Journal of Medical Science; The Report of the Medical Officer of Health for the Borough of Halifax; Talk; Food and Sanitation; The Review of Reviews; The Therapist; Modern Medicine; Proceedings of the Society for the Study of Inebriety; National Righteousness; The Religious Review of Reviews; The Review of the Churches; The Young Gentlewoman; Cornhill; The Whitehall Review; The Literary World; Pictures and Painters; London; Food and Sanitation; The Phonogram; The Provincial Medical Journal; The Charity Organization Review; Edinburgh Medical Missionary Society Quarterly Paper; The Medical Pioneer; The Technical World; A Thousand Miles in an Invalid's Coach, by Miss Robinson. Guy's Hospital Gazette; the Edinburgh Medical Journal; Cassell's Family Magazine; The Service of the King; the Clinical Journal; the International Journal of Surgery, Food, and Sanitation. The Religious Tract Society: The Sunday at Home, the Leisure Hour, the Boy's Own Paper, the Girl's Own Paper, Friendly Greetings, Light in the Home, Our Little Dots, the Cottager and Artisan, John Maggregor.

### MEDICAL VACANCIES AND APPOINTMENTS.

**APPOINTMENTS.**

A. Barker, M.D., Ch. D.Brux, L.R.C.S.I., appointed Visiting Medical Officer to the Nepean Cottage Hospital, Penrith, New South Wales. G. W. H. Bird, B.A., M.B., B.C.Cantab., L.R.C.P., M.R.C.S., reappointed Clinical Assistant in the Special Department for Diseases of the Skin, St. Thomas's Hospital. W. A. Bowring, L.R.C.P., M.R.C.S., appointed Senior Obstetric House Physician to St. Thomas's Hospital. A. B. Brockway, M.R.C.S., L.R.C.P.Lond., appointed Resident Surgeon to the Brisbane Hospital, Queensland. J. C. Buckley, M.B., Ch.B.Vict., appointed House Physician to the Hospital for Consumption and Diseases of the Chest, Brompton. Wm. Calwell, M.D., M.C., L.R.C.P.I., appointed House Physician to the Royal Hospital, Belfast. John Chadwick, M.R.C.S.Eng., L.S.A., appointed Medical Officer for the Butterworth District of the Rochdale Union. N. B. Clowes, L.R.C.P.Lond., M.R.C.S.Eng., appointed Medical Officer to the Reading Dispensary. Dr. Collins, appointed Medical Officer and Public Vaccinator for the Tow Law District of the Weardale Union. James John Young Dalgarno, M.B., C.M.Aberd., appointed Medical Officer to the Aberdeen General Dispensary. M. R. P. Dorman, M.A., M.B., B.C., D.P.H.Cantab., L.R.C.P., M.R.C.S., reappointed Non-resident House-Physician to St. Thomas's Hospital. J. H. Fisher, L.R.C.P., F.R.C.S., appointed Junior Obstetric House-Physician to St. Thomas's Hospital. F. J. Fletcher, L.S.A., appointed Medical Officer of the Burton Coggles District of the Grantham Union. H. J. Frederick, L.R.C.P., M.R.C.S., reappointed Clinical Assistant in the Special Department for Diseases of the Throat, St. Thomas's Hospital. L. D. Grover, L.R.C.P., reappointed Clinical Assistant in the Special Department for Diseases of the Ear, St. Thomas's Hospital. J. Harrison, M.D.Edin.,D.P.H., appointed Honorary Surgeon, North Shields, and Tynemouth Dispensary. T. W. Hicks, L.R.C.P., M.R.C.S., appointed Resident House-Physician to St. Thomas's Hospital. Dr. J. Holmes, appointed Medical Officer for the Penkridge District of the Cannock Union. W. H. Hughes, M.R.C.S.Eng., L.S.A., reappointed Medical Officer of Health for the Hinckley Urban Sanitary District. D. Huskie, M.A., M.B., C.M.Edin., appointed Parochial Medical Officer for Moffat. E. P. Isaacs, L.R.C.P., M.R.C.S., appointed Senior Ophthalmic House-Surgeon to St. Thomas's Hospital. C. S. Jaffé, L.R.C.P., M.R.C.S., appointed Clinical Assistant in the Special Department for Diseases of the Throat, St. Thomas's Hospital. P. Jamieson, M.A.Aberd., F.R.C.S.Edin., reappointed Medical Officer of Health and Police-Surgeon to the Peterhead Town Council. Dr. King, appointed Assistant Medical Officer of Health for the Barry and Cadoxton Urban Sanitary District. R. R. Law, B.A., M.B., B.C.Cantab, reappointed House-Surgeon to St. Thomas's Hospital. J. McOsar, L.R.C.P.Londr. M.R.C.S.Eng., appointed Medical Officer for the No. 9 (Chinnor) District of the Wycombe Union. A. R. O. Milton, L.R.C.P., M.R.C.S., reappointed Resident House-Physician to St. Thomas's Hospital. J. H. Murray-Aynsley, L.R.C.P.Lond., M.R.C.S., appointed Resident Medical Officer, Christchurch Hospital, N.Z.; also Surgeon to the Christ's College Rifles. P. Northcote, L.R.C.P., M.R.C.S., appointed Non-resident House-Physician to St. Thomas's Hospital. J. A. Offord, L.R.C.P., M.R.C.S., appointed Assistant Medical Officer, Dorset Asylum. H. L. Pearson, L.R.C.P.Lond., M.R.C.S.Eng., appointed Honorary Surgeon to the Wirral Hospital and Dispensary for Children. F. Perhouse, L.R.C.P., M.R.C.S., appointed Clinical Assistant in the Special Department for Diseases of the Skin, St. Thomas's Hospital. C. Planck, M.A.Cantab., L.R.C.P., M.R.C.S., appointed Assistant House-Surgeon to St. Thomas's Hospital. W. P. Purvis, M.B., B.Sc.Lond., L.R.C.P., M.R.C.S., reappointed House-Surgeon to St. Thomas's Hospital. W. Redpath, L.R.C.P., M.R.C.S., appointed Assistant House-Surgeon to St. Thomas's Hospital. J. Rowat, M.D.Glasg., D.P.H., appointed Medical Officer for the First District of the Highworth and Swindon Union. J. F. Rudall, M.B, B.S.Melb., M.R.C.S.Eng., appointed Junior Ophthalmic House-Surgeon to St. Thomas's Hospital. W. A. Rudd, M.B., appointed House-Surgson, Dorset County Hospital. C. H. Scott, M.B., Ch.B.Melb., appointed Visiting Medical Officer to the Nepean Cottage Hospital, Penrith, New South Wales. W. J. Shirlow, M.B., Syd., appointed Medical Officer to the Sydney Hospital, New South Wales. S. Sloan, M.D., appointed Consulting Physician to the Glasgow Samaritan

**ROYAL INFIRMARY, BRISTOL.**

There is a VACANCY for an HONORARY ASSISTANT PHYSICIAN. Applications and testimonials, by JUNE 10th, to the undersigned, who can forward a list of the Committee of Election.

J. F. SHEKLETON,

(862) Secretary and House Governor.

**ROYAL VICTORIA HOSPITAL, Bournemouth.**

### MEDICAL VACANCIES AND APPOINTMENTS—(continued)

Hospital. W. Woodley Stocker, M.R.C.S.Eng., L.R.C.P.Lond., appointed Medical Officer for the Willesden (No. 2) District of the Hendon Union. W. G. Sutcliffe, L.R.C.P., M.R.C.S., re-appointed House-Surgeon to St. Thomas's Hospital. W. L. Wainwright, L.R.C.P., M.R.C.S., reappointed House-Surgeon to St. Thomas's Hospital. A. Hastings Whicher, M.R.C.S.Eng., appointed Medical Officer of Health for the Midsomer Norton Urban Sanitary District. A. H. Woodcock, L.R.C.P., M.R.C.S., appointed Clinical Assistant in the Special Department for Diseases of the Ear, St. Thomas's Hospital. W. Wylie, M.D., C.M.Glasg., reappointed Medical Officer of Health for the Kirkby Lonsdale Urban Sanitary District.

### VACANCIES.

The following vacancies are announced this week, the amount of salary in each case being given after the name of the institution:

#### Resident Medical Officers.

Birmingham City Asylum. Resident Clinical Assistant. Board, residence, and washing provided. Applications to the Medical Superintendent.

City of Birmingham. Resident Medical Superintendent for the City Hospital for the Treatment of Small-pox and Scarlet Fever; doubly qualified, unmarried, and not more than 40 years of age. Salary £200 per annum, with furnished residence, coal, gas, attendance, washing, and rations. Applications, endorsed "Medical Superintendent," to the Clerk, Health Committee, Council House, Birmingham, by June 16th.

East London Hospital for Children, Glamis Road, Shadwell, E. House-Surgeon. Board and lodging provided. Applications to the Secretary by July 6th.

General Infirmary, Northampton. House-Surgeon; doubly qualified, unmarried, and not under 23 years of age. Salary £125 per annum, with furnished apartments, board, attendance, and washing. The Assistant House-Surgeon is a candidate, and in the event of his appointment, the Court will proceed to the election of an Assistant House-Surgeon. Salary £80 per annum, furnished apartments board, attendance, and washing. Applications to the Secretary by June 10th.

Macclesfield General Infirmary. Junior House-Surgeon; doubly qualified. Salary £70 per annum, with board and residence. Applications to the Chairman of the House Committee by June 15th.

Norfolk County Asylum, Thorpe, Norwich. Temporary Assistant Medical Officer. Board, lodging, and washing provided. Applications to the Medical Superintendent.

Royal Albert Hospital, Devonport. Assistant House-Surgeon. Board, lodging, and washing provided. Applications to the Chairman of Medical Committee by June 21st.

Royal Berks Hospital. Assistant House-Surgeon. Salary £40 per annum, with board and lodging. Applications to the Secretary by June 13th.

Royal Free Hospital, Gray's Inn Road, W.C. Junior Resident Medical Officer. House-Surgeon; doubly qualified. Appointment for six months. Board, residence, and washing provided. Applications to the Secretary by June 19th.

West London Hospital, Hammersmith Road, W. House-Physician and House-Surgeon. Board and lodging provided. Applications to R. J. Gilbert, Secretary-Superintendent, by June 21st.

#### Non-Resident Medical Officers.

Faringdon Union, Faringdon. Medical Officer and Public Vaccinator for the Buckland District. Salary £126 per annum, with extra fees allowed by the Local Government Board. Applications to the Clerk of the Guardians by June 13th.

Metropolitan Hospital, Kingsland Road, N.E. Ophthalmic Surgeon. Must be F.R.C.S.Eng. Applications to Charles H. Byers, Secretary, by June 9th.

West London Hospital, Hammersmith Road, W. Surgeon. In view of one of the Assistant Surgeons being elected, applications are invited for Assistant Surgeon. Applications to R. J. Gilbert, Secretary-Superintendent, by June 21st.

Westminster Hospital, Broad Sanctuary, S.W. Assistant Obstetric Physician. Applications to the Secretary.

North-Eastern Hospital for Children, Hackney Road, N.E. Junior House-Surgeon; doubly qualified. Appointment for six months. Salary at the rate of £60 per annum. Applications to Alfred Nixon, Secretary, 27, Clement's Lane, E.C., by June 10th.

Royal Infirmary, Bristol. Honorary Assistant Physician. Applications and testimonials to J. F. Shekleton, Secretary and House Governor (who can forward a list of the Committee of Election), by June 10th.

Royal Victoria Hospital, Bournemouth. Ophthalmic Surgeon. Applications to the Secretary by July 1st; and a House Surgeon-Secretary. Salary £100 per annum, and board for the latter. Applications to the Chairman of Committee by July 1st.

Sunderland Infirmary. Honorary Medical Officer to the Convalescent House at Heatherdene, Harrogate; doubly qualified. Applications to the Chairman of the Medical Board by June 6th.

University of London, Burlington Gardens, W. Examiner in Surgery. Salary £200 per annum. Applications to the Registrar by June 24th.

Victoria Hospital for Sick Children, Queen's Road, Chelsea, S.W. Surgeon on the in-patient staff and Surgeon on the out-patient staff. Appointment for five years. Applications to Commander Blount, R.N., Secretary, by June 17th.

